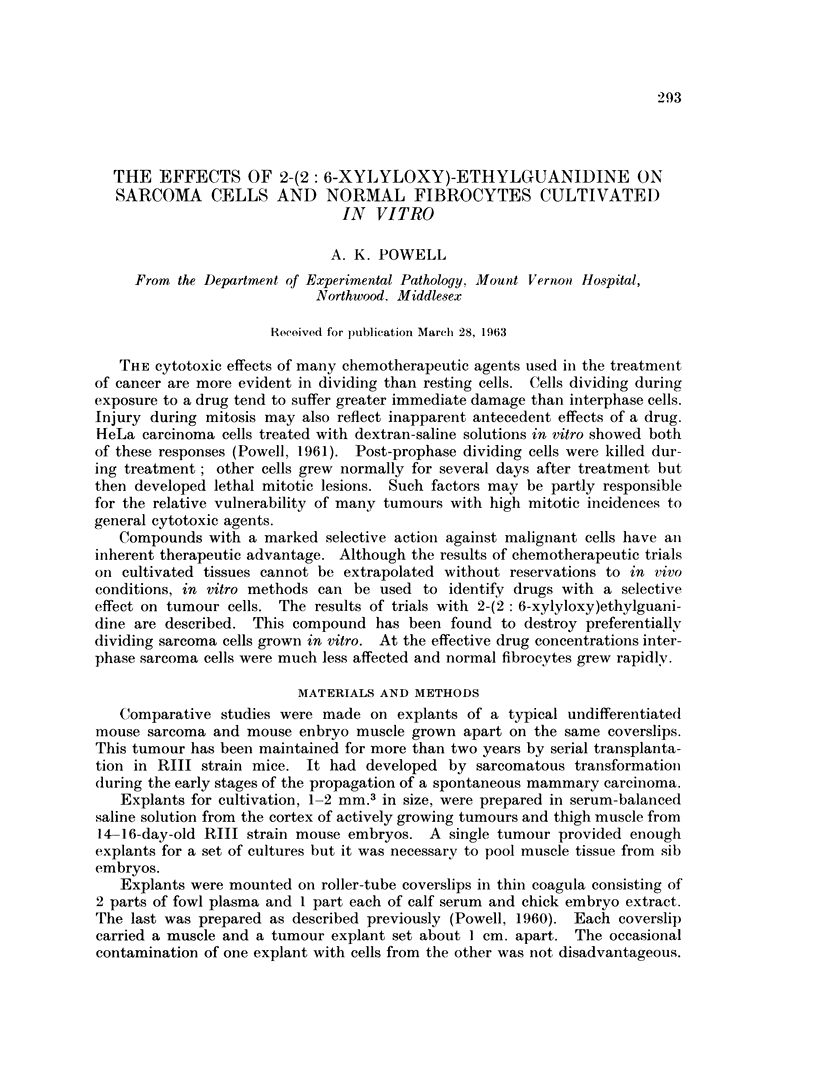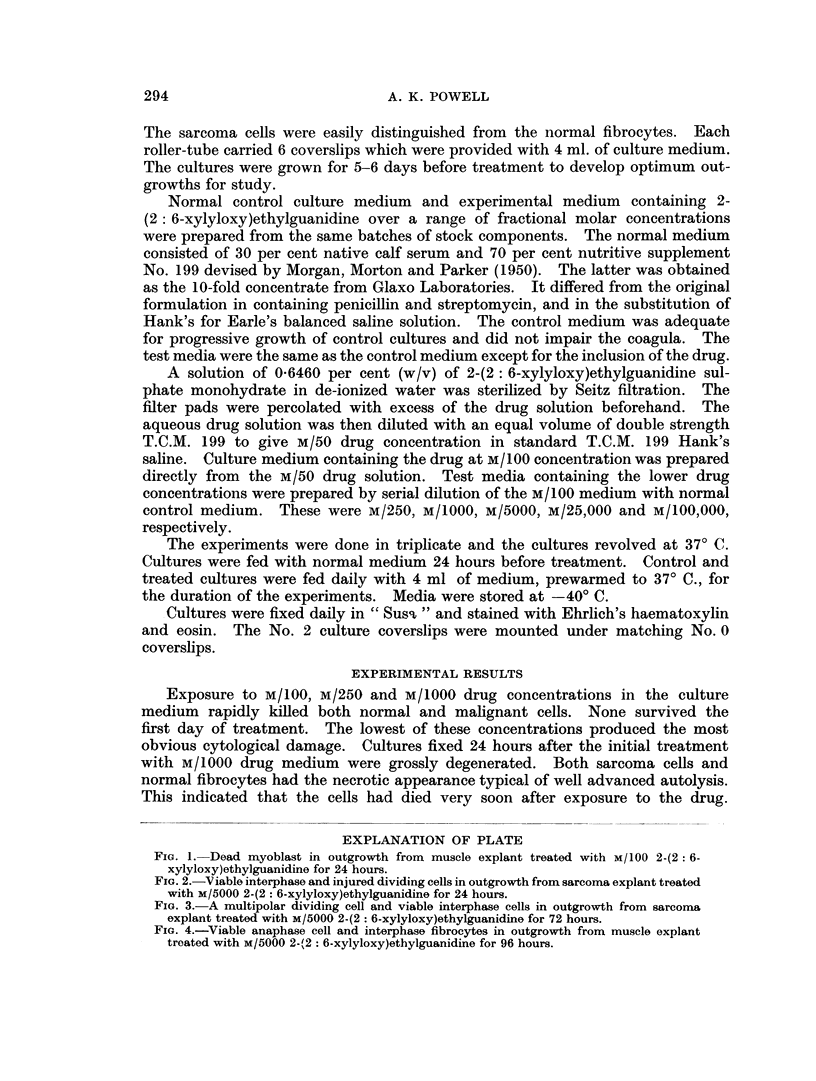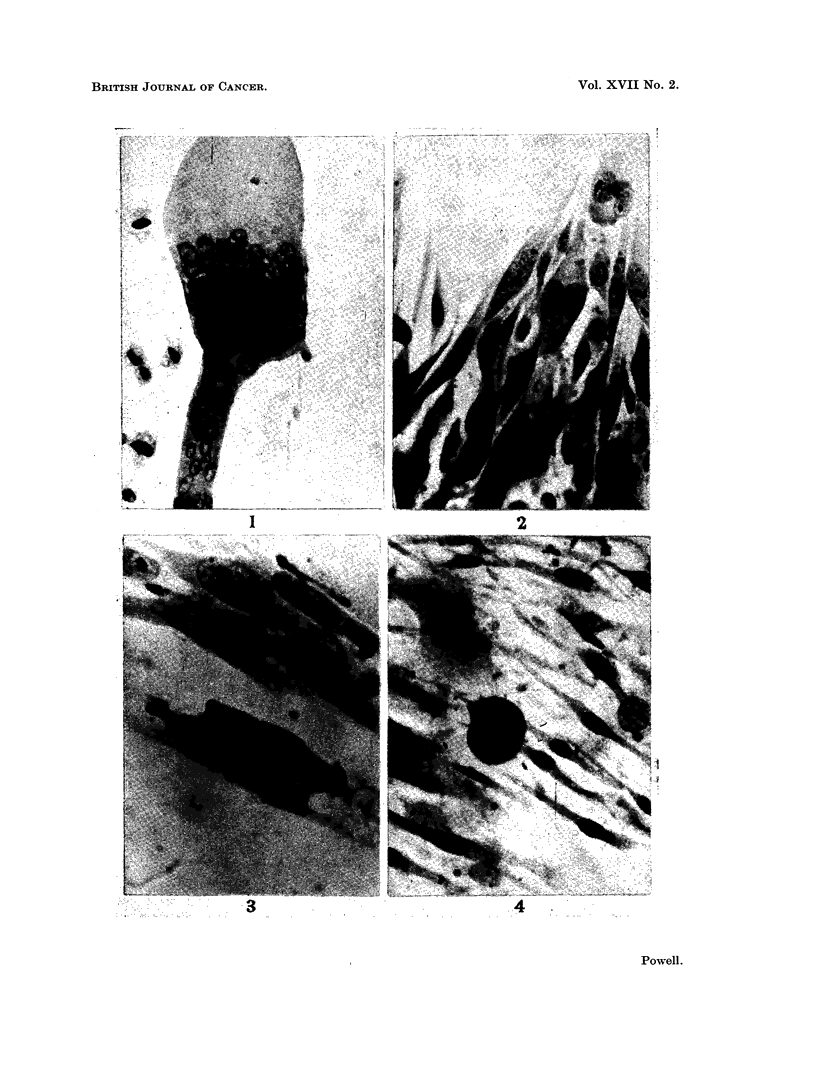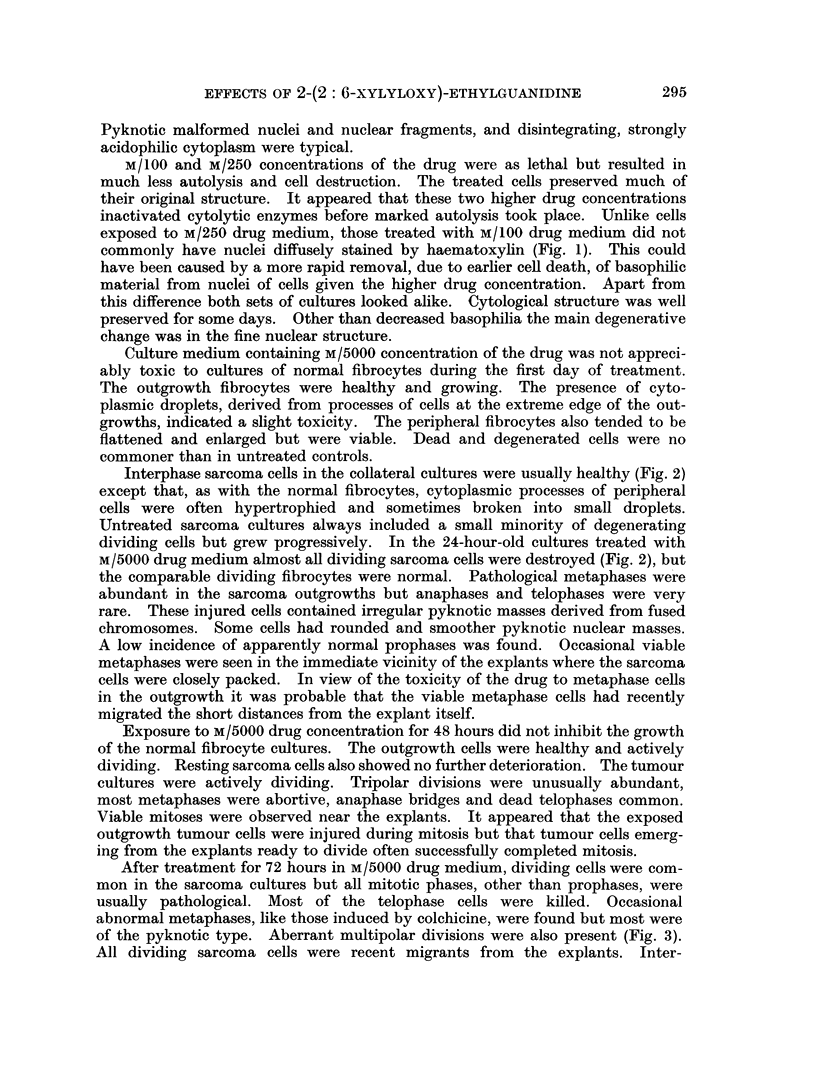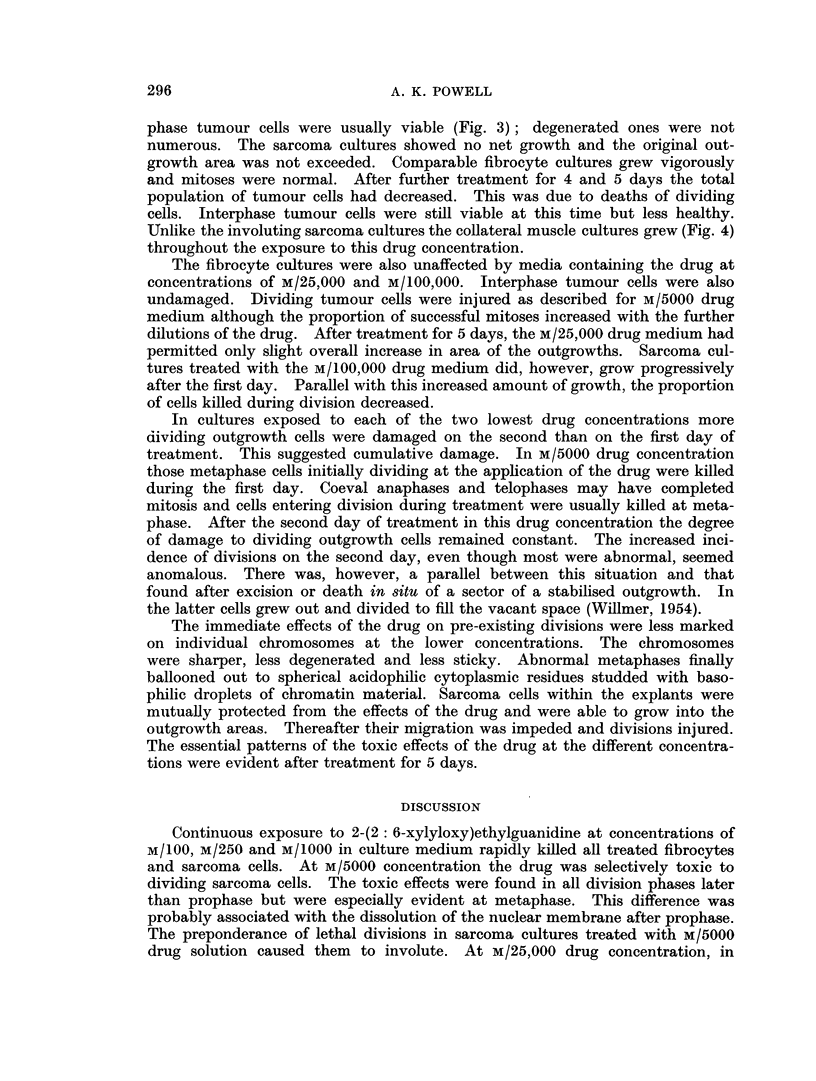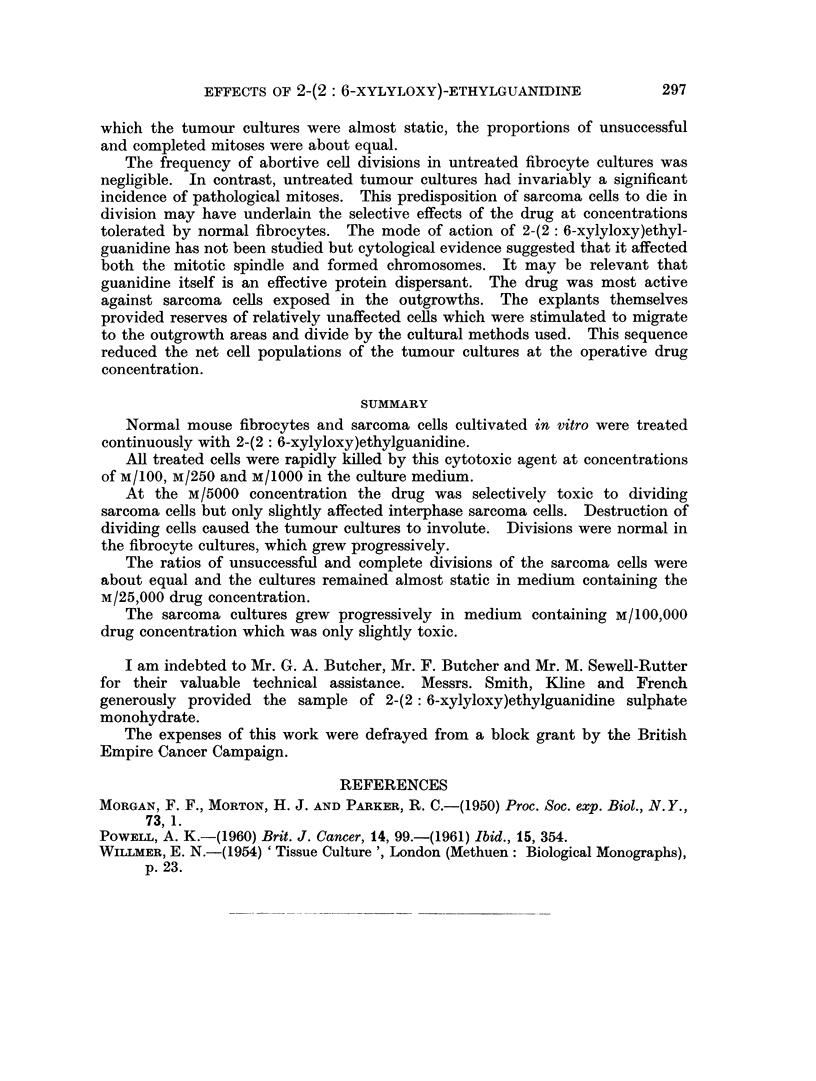# The Effects of 2-(2:6-Xylyloxy)-Ethylguanidine on Sarcoma Cells and Normal Fibrocytes Cultivated In Vitro

**DOI:** 10.1038/bjc.1963.42

**Published:** 1963-06

**Authors:** A. K. Powell

## Abstract

**Images:**


### THE EFFECTS OF 2-(2 : 6-XYLYLOXY)-ETHYLGUANIDINE ON SARCOMA CELLS AND NORMAL FIBROCYTES CULTIVATED *IN VITRO*

A. K. POWELL

*From the Department of Experimental Pathology, Mount Vernon Hospital, Northwood, Middlesex*

Received for publication March 28, 1963

THE cytotoxic effects of many chemotherapeutic agents used in the treatment of cancer are more evident in dividing than resting cells. Cells dividing during exposure to a drug tend to suffer greater immediate damage than interphase cells. Injury during mitosis may also reflect inapparent antecedent effects of a drug. HeLa carcinoma cells treated with dextran-saline solutions *in vitro* showed both of these responses (Powell, 1961). Post-prophase dividing cells were killed during treatment ; other cells grew normally for several days after treatment but then developed lethal mitotic lesions. Such factors may be partly responsible for the relative vulnerability of many tumours with high mitotic incidences to general cytotoxic agents.

Compounds with a marked selective action against malignant cells have an inherent therapeutic advantage. Although the results of chemotherapeutic trials on cultivated tissues cannot be extrapolated without reservations to *in vivo* conditions, *in vitro* methods can be used to identify drugs with a selective effect on tumour cells. The results of trials with 2-(2 : 6-xylyloxy)ethylguanidine are described. This compound has been found to destroy preferentially dividing sarcoma cells grown *in vitro*. At the effective drug concentrations interphase sarcoma cells were much less affected and normal fibrocytes grew rapidly.

### MATERIALS AND METHODS

Comparative studies were made on explants of a typical undifferentiated mouse sarcoma and mouse enbryo muscle grown apart on the same coverslips. This tumour has been maintained for more than two years by serial transplantation in RIII strain mice. It had developed by sarcomatous transformation during the early stages of the propagation of a spontaneous mammary carcinoma.

Explants for cultivation, 1–2 $mm.^3$ in size, were prepared in serum-balanced saline solution from the cortex of actively growing tumours and thigh muscle from 14–16-day-old RIII strain mouse embryos. A single tumour provided enough explants for a set of cultures but it was necessary to pool muscle tissue from sib embryos.

Explants were mounted on roller-tube coverslips in thin coagula consisting of 2 parts of fowl plasma and 1 part each of calf serum and chick embryo extract. The last was prepared as described previously (Powell, 1960). Each coverslip carried a muscle and a tumour explant set about 1 cm. apart. The occasional contamination of one explant with cells from the other was not disadvantageous.

The sarcoma cells were easily distinguished from the normal fibrocytes. Each roller-tube carried 6 coverslips which were provided with 4 ml. of culture medium. The cultures were grown for 5–6 days before treatment to develop optimum outgrowths for study.

Normal control culture medium and experimental medium containing 2-(2 : 6-xylyloxy)ethylguanidine over a range of fractional molar concentrations were prepared from the same batches of stock components. The normal medium consisted of 30 per cent native calf serum and 70 per cent nutritive supplement No. 199 devised by Morgan, Morton and Parker (1950). The latter was obtained as the 10-fold concentrate from Glaxo Laboratories. It differed from the original formulation in containing penicillin and streptomycin, and in the substitution of Hank's for Earle's balanced saline solution. The control medium was adequate for progressive growth of control cultures and did not impair the coagula. The test media were the same as the control medium except for the inclusion of the drug.

A solution of 0·6460 per cent (w/v) of 2-(2 : 6-xylyloxy)ethylguanidine sulphate monohydrate in de-ionized water was sterilized by Seitz filtration. The filter pads were percolated with excess of the drug solution beforehand. The aqueous drug solution was then diluted with an equal volume of double strength T.C.M. 199 to give M/50 drug concentration in standard T.C.M. 199 Hank's saline. Culture medium containing the drug at M/100 concentration was prepared directly from the M/50 drug solution. Test media containing the lower drug concentrations were prepared by serial dilution of the M/100 medium with normal control medium. These were M/250, M/1000, M/5000, M/25,000 and M/100,000, respectively.

The experiments were done in triplicate and the cultures revolved at 37° C. Cultures were fed with normal medium 24 hours before treatment. Control and treated cultures were fed daily with 4 ml of medium, prewarmed to 37° C., for the duration of the experiments. Media were stored at —40° C.

Cultures were fixed daily in " Susa " and stained with Ehrlich's haematoxylin and eosin. The No. 2 culture coverslips were mounted under matching No. 0 coverslips.

### EXPERIMENTAL RESULTS

Exposure to M/100, M/250 and M/1000 drug concentrations in the culture medium rapidly killed both normal and malignant cells. None survived the first day of treatment. The lowest of these concentrations produced the most obvious cytological damage. Cultures fixed 24 hours after the initial treatment with M/1000 drug medium were grossly degenerated. Both sarcoma cells and normal fibrocytes had the necrotic appearance typical of well advanced autolysis. This indicated that the cells had died very soon after exposure to the drug.

---

### EXPLANATION OF PLATE

FIG. 1.—Dead myoblast in outgrowth from muscle explant treated with M/100 2-(2 : 6-xylyloxy)ethylguanidine for 24 hours.

FIG. 2.—Viable interphase and injured dividing cells in outgrowth from sarcoma explant treated with M/5000 2-(2 : 6-xylyloxy)ethylguanidine for 24 hours.

FIG. 3.—A multipolar dividing cell and viable interphase cells in outgrowth from sarcoma explant treated with M/5000 2-(2 : 6-xylyloxy)ethylguanidine for 72 hours.

FIG. 4.—Viable anaphase cell and interphase fibrocytes in outgrowth from muscle explant treated with M/5000 2-(2 : 6-xylyloxy)ethylguanidine for 96 hours.

1

2

3

4

Pyknotic malformed nuclei and nuclear fragments, and disintegrating, strongly acidophilic cytoplasm were typical.

M/100 and M/250 concentrations of the drug were as lethal but resulted in much less autolysis and cell destruction. The treated cells preserved much of their original structure. It appeared that these two higher drug concentrations inactivated cytolytic enzymes before marked autolysis took place. Unlike cells exposed to M/250 drug medium, those treated with M/100 drug medium did not commonly have nuclei diffusely stained by haematoxylin (Fig. 1). This could have been caused by a more rapid removal, due to earlier cell death, of basophilic material from nuclei of cells given the higher drug concentration. Apart from this difference both sets of cultures looked alike. Cytological structure was well preserved for some days. Other than decreased basophilia the main degenerative change was in the fine nuclear structure.

Culture medium containing M/5000 concentration of the drug was not appreciably toxic to cultures of normal fibrocytes during the first day of treatment. The outgrowth fibrocytes were healthy and growing. The presence of cytoplasmic droplets, derived from processes of cells at the extreme edge of the outgrowths, indicated a slight toxicity. The peripheral fibrocytes also tended to be flattened and enlarged but were viable. Dead and degenerated cells were no commoner than in untreated controls.

Interphase sarcoma cells in the collateral cultures were usually healthy (Fig. 2) except that, as with the normal fibrocytes, cytoplasmic processes of peripheral cells were often hypertrophied and sometimes broken into small droplets. Untreated sarcoma cultures always included a small minority of degenerating dividing cells but grew progressively. In the 24-hour-old cultures treated with M/5000 drug medium almost all dividing sarcoma cells were destroyed (Fig. 2), but the comparable dividing fibrocytes were normal. Pathological metaphases were abundant in the sarcoma outgrowths but anaphases and telophases were very rare. These injured cells contained irregular pyknotic masses derived from fused chromosomes. Some cells had rounded and smoother pyknotic nuclear masses. A low incidence of apparently normal prophases was found. Occasional viable metaphases were seen in the immediate vicinity of the explants where the sarcoma cells were closely packed. In view of the toxicity of the drug to metaphase cells in the outgrowth it was probable that the viable metaphase cells had recently migrated the short distances from the explant itself.

Exposure to M/5000 drug concentration for 48 hours did not inhibit the growth of the normal fibrocyte cultures. The outgrowth cells were healthy and actively dividing. Resting sarcoma cells also showed no further deterioration. The tumour cultures were actively dividing. Tripolar divisions were unusually abundant, most metaphases were abortive, anaphase bridges and dead telophases common. Viable mitoses were observed near the explants. It appeared that the exposed outgrowth tumour cells were injured during mitosis but that tumour cells emerging from the explants ready to divide often successfully completed mitosis.

After treatment for 72 hours in M/5000 drug medium, dividing cells were common in the sarcoma cultures but all mitotic phases, other than prophases, were usually pathological. Most of the telophase cells were killed. Occasional abnormal metaphases, like those induced by colchicine, were found but most were of the pyknotic type. Aberrant multipolar divisions were also present (Fig. 3). All dividing sarcoma cells were recent migrants from the explants. Interphase tumour cells were usually viable (Fig. 3); degenerated ones were not numerous. The sarcoma cultures showed no net growth and the original outgrowth area was not exceeded. Comparable fibrocyte cultures grew vigorously and mitoses were normal. After further treatment for 4 and 5 days the total population of tumour cells had decreased. This was due to deaths of dividing cells. Interphase tumour cells were still viable at this time but less healthy. Unlike the involuting sarcoma cultures the collateral muscle cultures grew (Fig. 4) throughout the exposure to this drug concentration.

The fibrocyte cultures were also unaffected by media containing the drug at concentrations of M/25,000 and M/100,000. Interphase tumour cells were also undamaged. Dividing tumour cells were injured as described for M/5000 drug medium although the proportion of successful mitoses increased with the further dilutions of the drug. After treatment for 5 days, the M/25,000 drug medium had permitted only slight overall increase in area of the outgrowths. Sarcoma cultures treated with the M/100,000 drug medium did, however, grow progressively after the first day. Parallel with this increased amount of growth, the proportion of cells killed during division decreased.

In cultures exposed to each of the two lowest drug concentrations more dividing outgrowth cells were damaged on the second than on the first day of treatment. This suggested cumulative damage. In M/5000 drug concentration those metaphase cells initially dividing at the application of the drug were killed during the first day. Coeval anaphases and telophases may have completed mitosis and cells entering division during treatment were usually killed at metaphase. After the second day of treatment in this drug concentration the degree of damage to dividing outgrowth cells remained constant. The increased incidence of divisions on the second day, even though most were abnormal, seemed anomalous. There was, however, a parallel between this situation and that found after excision or death *in situ* of a sector of a stabilised outgrowth. In the latter cells grew out and divided to fill the vacant space (Willmer, 1954).

The immediate effects of the drug on pre-existing divisions were less marked on individual chromosomes at the lower concentrations. The chromosomes were sharper, less degenerated and less sticky. Abnormal metaphases finally ballooned out to spherical acidophilic cytoplasmic residues studded with basophilic droplets of chromatin material. Sarcoma cells within the explants were mutually protected from the effects of the drug and were able to grow into the outgrowth areas. Thereafter their migration was impeded and divisions injured. The essential patterns of the toxic effects of the drug at the different concentrations were evident after treatment for 5 days.

### DISCUSSION

Continuous exposure to 2-(2 : 6-xylyloxy)ethylguanidine at concentrations of M/100, M/250 and M/1000 in culture medium rapidly killed all treated fibrocytes and sarcoma cells. At M/5000 concentration the drug was selectively toxic to dividing sarcoma cells. The toxic effects were found in all division phases later than prophase but were especially evident at metaphase. This difference was probably associated with the dissolution of the nuclear membrane after prophase. The preponderance of lethal divisions in sarcoma cultures treated with M/5000 drug solution caused them to involute. At M/25,000 drug concentration, in which the tumour cultures were almost static, the proportions of unsuccessful and completed mitoses were about equal.

The frequency of abortive cell divisions in untreated fibrocyte cultures was negligible. In contrast, untreated tumour cultures had invariably a significant incidence of pathological mitoses. This predisposition of sarcoma cells to die in division may have underlain the selective effects of the drug at concentrations tolerated by normal fibrocytes. The mode of action of 2-(2 : 6-xylyloxy)ethylguanidine has not been studied but cytological evidence suggested that it affected both the mitotic spindle and formed chromosomes. It may be relevant that guanidine itself is an effective protein dispersant. The drug was most active against sarcoma cells exposed in the outgrowths. The explants themselves provided reserves of relatively unaffected cells which were stimulated to migrate to the outgrowth areas and divide by the cultural methods used. This sequence reduced the net cell populations of the tumour cultures at the operative drug concentration.

SUMMARY

Normal mouse fibrocytes and sarcoma cells cultivated *in vitro* were treated continuously with 2-(2 : 6-xylyloxy)ethylguanidine.

All treated cells were rapidly killed by this cytotoxic agent at concentrations of M/100, M/250 and M/1000 in the culture medium.

At the M/5000 concentration the drug was selectively toxic to dividing sarcoma cells but only slightly affected interphase sarcoma cells. Destruction of dividing cells caused the tumour cultures to involute. Divisions were normal in the fibrocyte cultures, which grew progressively.

The ratios of unsuccessful and complete divisions of the sarcoma cells were about equal and the cultures remained almost static in medium containing the M/25,000 drug concentration.

The sarcoma cultures grew progressively in medium containing M/100,000 drug concentration which was only slightly toxic.

I am indebted to Mr. G. A. Butcher, Mr. F. Butcher and Mr. M. Sewell-Rutter for their valuable technical assistance. Messrs. Smith, Kline and French generously provided the sample of 2-(2 : 6-xylyloxy)ethylguanidine sulphate monohydrate.

The expenses of this work were defrayed from a block grant by the British Empire Cancer Campaign.

### REFERENCES

MORGAN, F. F., MORTON, H. J. AND PARKER, R. C.—(1950) *Proc. Soc. exp. Biol., N.Y.*, **73**, 1.

POWELL, A. K.—(1960) *Brit. J. Cancer*, **14**, 99.—(1961) *Ibid.*, **15**, 354.

WILLMER, E. N.—(1954) ' Tissue Culture ', London (Methuen : Biological Monographs), p. 23.